# A Novel Approach for Rehabilitation of a Subtotal Maxillectomy Patient with Immediately Loaded Basal Implant-Supported Prosthesis: 4 Years Follow-Up

**DOI:** 10.1155/2020/9650164

**Published:** 2020-02-06

**Authors:** Motaz Osman, Abdelnasir G. Ahmad, Fadia Awadalkreem

**Affiliations:** ^1^Department of Implantology, Khartoum Teaching Dental Hospital, Federal Ministry of Heath, Khartoum, Sudan; ^2^Department of Oral and Maxillofacial Surgery, Faculty of Dentistry, International University of Africa, Khartoum, Sudan; ^3^Department of Oral Rehabilitation, Prosthodontic Division, Faculty of Dentistry, University of Khartoum, Sudan

## Abstract

The prosthetic rehabilitation of maxillary defect can be achieved successfully by using an implant-supported prosthesis. The use of remote bony areas such as the zygomatic bone in cases of large defects provides an innovative substitute for free vascularized osteocutaneous flaps and the solution to flap failures. This report describes the rehabilitation of a 22-year-old female with a subtotal maxillectomy using an immediately loaded basal implant-supported prosthesis. Four basal cortical screw implants (BCS®) are inserted; 1 on the contralateral nasal floor, 2 implants in the pterygoid plates, and the last in the zygomatic bone using cone beam computed tomography scans. The prosthesis was constructed and cemented in 3 days. The surgical and prosthetic procedures were performed without any obstacles. After 4 years in function, the patient was highly satisfied with the treatment as it improved her mastication, speech, aesthetic and returned her self-esteem. To our knowledge, this is the first clinical report detailing the use of basal implant-retained obturator in a subtotal maxillectomy patient.

## 1. Introduction

The maxilla is a fundamental structure in the face that plays a critical role in esthetics, speech, swallowing, and mastication [1]. It separates the oral, antral, and orbital cavities and provides support to many vital structures such as the lower eyelids, cheeks, lips, and nose [1]. Any maxillary defect regardless of its size affects speech, swallowing, and mastication; may result in cosmetic disfigurement; and compromised the patient's quality of life [1, 2]. Reconstruction of maxillary defects is one of the most challenging works the maxillofacial surgeons and prosthodontists are facing [1, 2].

The aims of maxillary defect reconstruction should include closure of the defect, separation of the oral cavity from the sinus and nasal cavities [2–4], maintenance of orbital content support, preservation of eyelid functions, cleared nasal airways, replacement of masticatory units, improving esthetics, and restoring normal or near to normal patient life [2–5]. Subtotal maxillectomy is a term used to define “any maxillary resection involving the removal of at least two walls, including the floor of the antrum (the hard palate) with the exception of the posterior wall” [6]. Reconstruction of subtotal maxillectomy can be achieved either through surgical reconstruction, prosthetic reconstruction (obturator), or a combination of both techniques [2–4].

Many surgical techniques such as local/regional flaps [2, 3], soft tissue, and/or bone free flaps have been reported although their use alone in massive defect reconstruction was limited [3].

Consequently, large maxillary defects can be repaired using obturators or a combination of both techniques [1, 3].

The main advantages of an obturator are shorter treatment time, reduced cost, and easy visualization of the maxillectomy cavity [2, 3]. However, obturator therapy has many disadvantages, including lack of retention in cases of large defects, reduction in supportive dentition, discomfort associated with prosthesis wear, the potential of hypernasal speech, and regurgitation of foods and liquids into the nasal cavity in cases of an inadequate seal [2, 3]. In addition, other drawbacks include the inconvenience of prosthesis removal and cleaning to maintain defect hygiene and the periodic need for prosthesis adjustments following healing and bone remodeling [2].

Many methods of maxillofacial prosthesis retention and support have been reported, such as engagement of the remaining teeth, bony anatomic undercuts, and lateral scar band [2–4].

Unfortunately, in A very large defect and/or when primary closure of soft tissue defects is achieved immediately after resection, the gingivobuccal sulcus is reduced, and the undercuts required for prosthesis retention are deficient or even eliminated [3]. In such cases, implants can improve obturator retention, support, and stability and therefore improve the patient's quality of life [7–13].

Following maxillectomy, a limited amount of maxillary bone remains; therefore, implant placement utilizes the use of more distant sites [7–12] such as the zygomatic bone [8, 10, 11] and pterygoid bone [12]. In 1989, Bidra et al. and Tulasne [12, 13] introduced the Tubero-pterygoid implants, which are inserted through the maxillary tuberosity in an oblique direction proceeding deeply to the pterygoid plate. On the other hand, zygomatic implants were firstly described by Branemark [14] in 1989. Several authors [3, 8, 10, 11, 15, 16] have reported the use of zygomatic implants to eliminate the need for bone grafting, reduce the risk of implant failure, and shorten treatment time.

Basal Cortical Screw (BCS ®) implants are basal implants with specific characteristics [17–19].

They are designed in an extended length, up to 55 mm, to be inserted from the crestal direction and anchored securely (osseofixated) into the remaining remote basal bone such as the pterygoid plate of the sphenoid and zygomatic bones providing a 2nd or even 3rd cortex engagement [17, 18]. The insertion of conventional implants into the pterygoid and zygomatic bones has been previously described [10–16]. However, evidence concerning the use of basal implants in these areas is limited.

This clinical report describes a successful case of maxillary defect reconstruction via fixed implant-supported prosthesis using basal implants inserted into the nasal floor, zygomatic, and pterygoid plate bones.

## 2. Case Presentation

**Figure 1:**
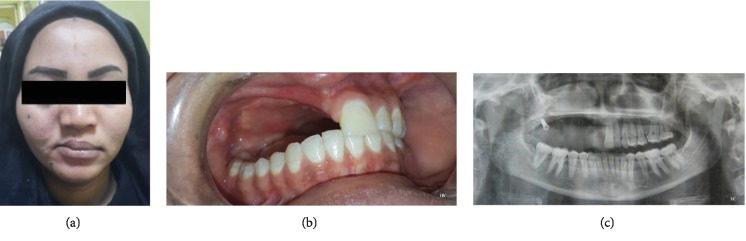
(a) Patient's frontal view at the time of presentation showing depressed check. (b) Patient's intraoral view showing right partial maxillectomy after soft tissue healing. (c) A preoperative panoramic view showing previously placed implant at the right tuberosity area.

A 22-year-old female patient presented at the Khartoum Teaching Dental Hospital with a swelling on the right side of her face. She was diagnosed with odontogenic myxoma. Subtotal maxillectomy was performed, and the intraoral defect was closed using soft tissue approximation (Figures 1(a) and 1(b)). One year later, the patient returned with esthetic complaints and reported masticatory inefficiency; she requested a fixed prosthesis. The patient was very depressed. During the previous 6 months, she visited a dentist who inserted one implant in the area of the maxillary tuberosity to construct an implant-supported prosthesis, but unfortunately, the prosthesis was not retained. This treatment failure had led to further deterioration of her emotional status.

A multidisciplinary team was formed, including an expert oral maxillofacial surgeon and a removable and fixed prosthodontists to avoid any technical complication. Preoperative radiographs (digital panorama and cone beam CT) was performed in order to evaluate the treatment options and identify optimal bone areas for implant anchorage (Figure 1(c)). The formulated treatment plan included the construction of immediately loaded fixed basal implant-supported obturator. The treatment plan was fully discussed with the patient, and informed consent was obtained.

**Figure 2:**
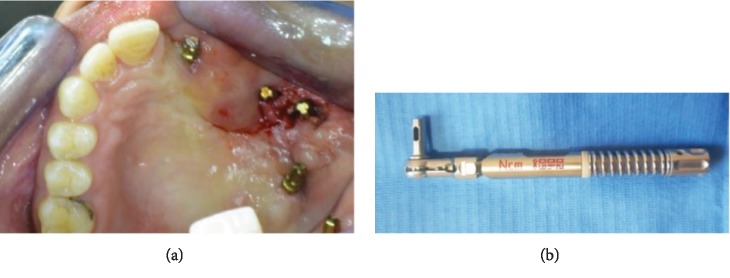
(a) Patient's intraoral view showing implant's distribution (image using mirror). (b) Tool used for implant bending.

Four BCS® implants (Dr. Ihde Dental, Switzerland) were inserted using local anesthesia (2% lidocaine with epinephrine 1 : 100,000) and flapless technique. Implant anchored are as follows: one implant in the contralateral nasal floor, two pterygoid implants, and one implant in the zygomatic bone with 3.5 mm width and 29, 32, 35, and 35 mm length, respectively. (Figure 2(a)).

More than 60 N cm insertion torque was obtained [20]. The anterior implant (inserted in the contralateral nasal floor) was bent using a special bending tool provided by the implant's company for more favorable prosthetic orientation [19, 21] (Figure 2(b)).

**Figure 3:**
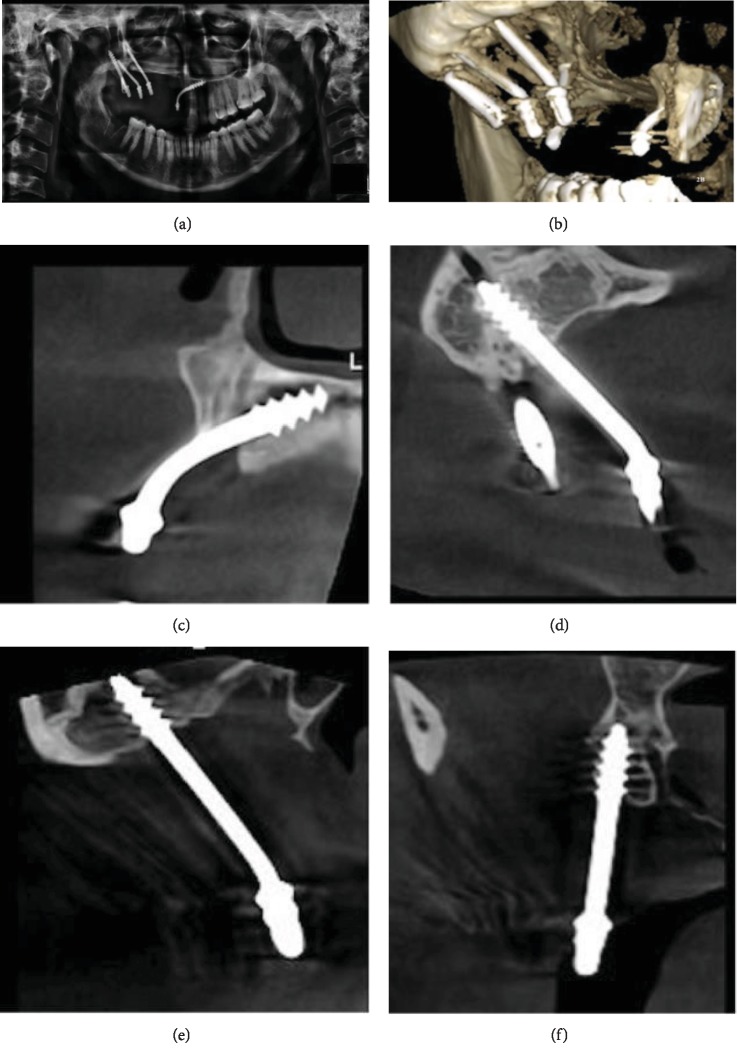
(a) Postoperative panoramic view showing that the overerupted 47 had been extracted to avoid unwanted and noncontrollable further eruption, which could damage the implant's integration through mechanical overload. In addition to the lake of the opposing artificial teeth occlusion (the prosthesis has only an upper first molar) to eliminate the cantilever force and to reduce and ensure better force distribution. (b–f) A cone beam computer tomography showing a 3-dimension view for the implants' positions; one in the contralateral nasal floor, two implants at the pterygoid plates, and one implant at the zygomatic bone.

Postoperative panoramic and cone beam computed tomography views were performed to verify the implant's positions (Figures 3(a)–3(f)).

**Figure 4:**
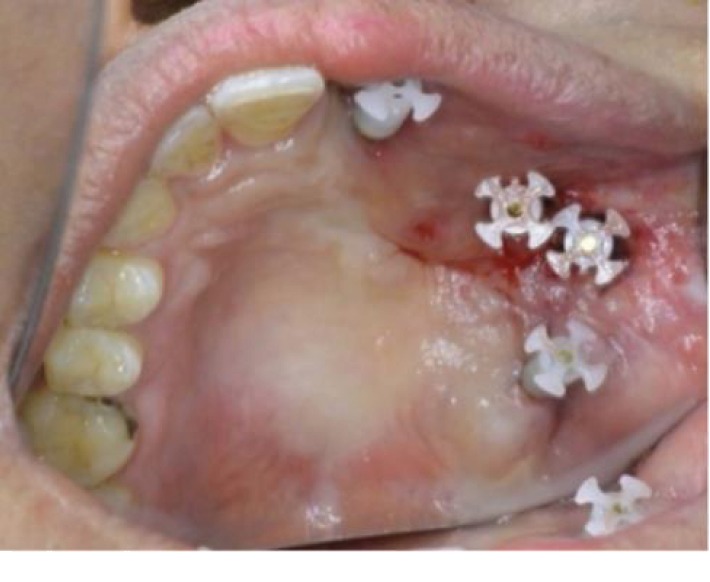
Image showing impression copping attached to the abutments head (image using mirror).

Impression coping was attached to the implants' head (Figure 4). An impression was taken using monophase vinyl polysiloxane (VPS, Ivoclar Vivadent AG). Amoxicillin and clavulanate potassium 1 mg (Megamox, HIKMA), diclofenac potassium 50 mg (Rapidus, Tabuk), and xylometazoline adult nasal drops (Otrivin, GlaxoSmithKline) were prescribed.

**Figure 5:**
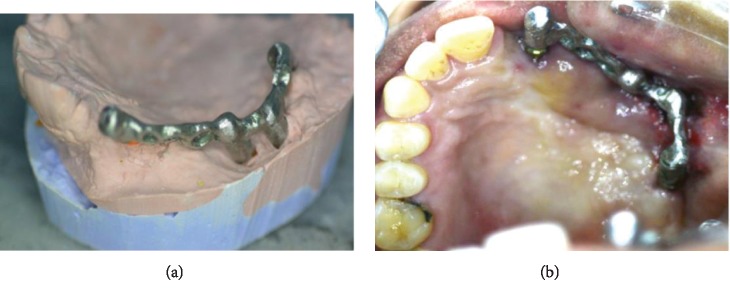
(a) Image showing the metal framework in the cast. (b) Image showing the metal framework try-in inside the patient's mouth (image using a mirror).

One day later, a metal framework try-in was performed, followed by silicone jaw relation (Figures 5(a) and 5(b)).

**Figure 6:**
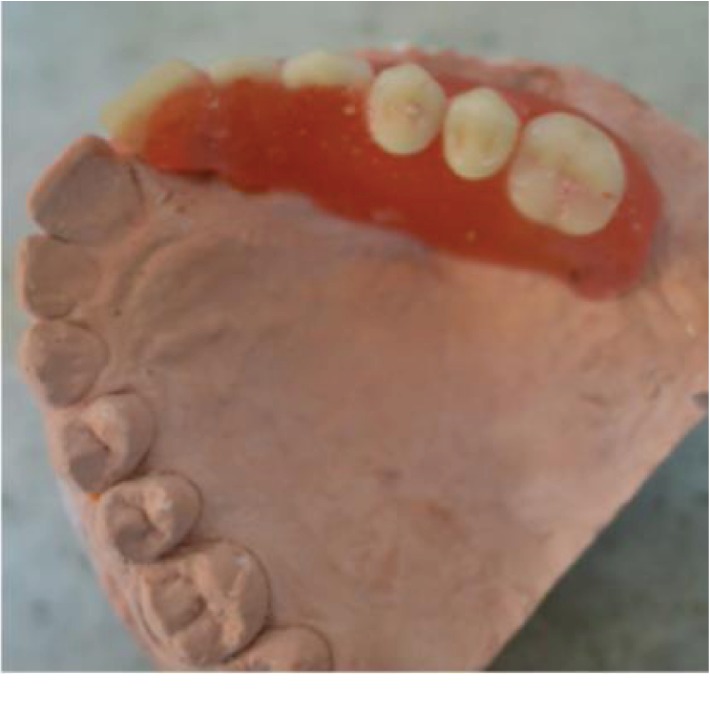
Image showing the wax try-in in the cast.

Both acrylic teeth and veneer material were added, and a wax try-in was done to verify the patient esthetic and occlusion (Figure 6).

**Figure 7:**
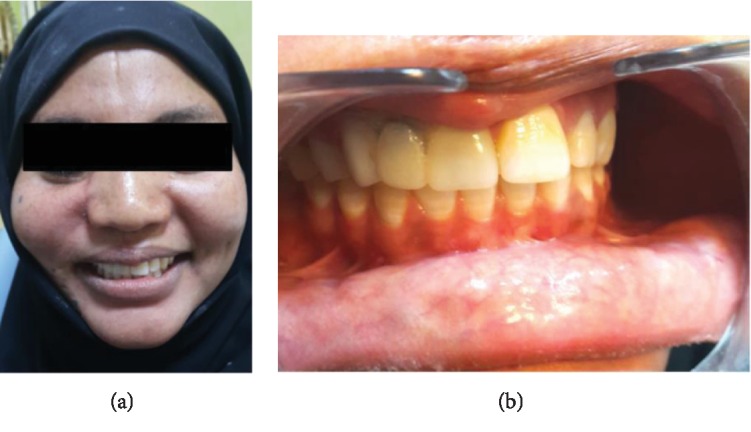
(a) Patient's frontal view showing the clinical outcome after the final insertion of the fixed basal implant-supported prosthesis. (b) Intraoral view showing the clinical outcome after the final insertion of the fixed basal implant-supported prosthesis.

The labial and palatal flanges were shortened (not extended to the full depth of the sulcus, i.e., hygienic design) for hygienic purposes. On the third day, the final prosthesis was inserted and cemented using Fuji cement (GC Fuji I Luting Cement, Japan) (Figures 7(a) and 7(b)).

**Figure 8:**
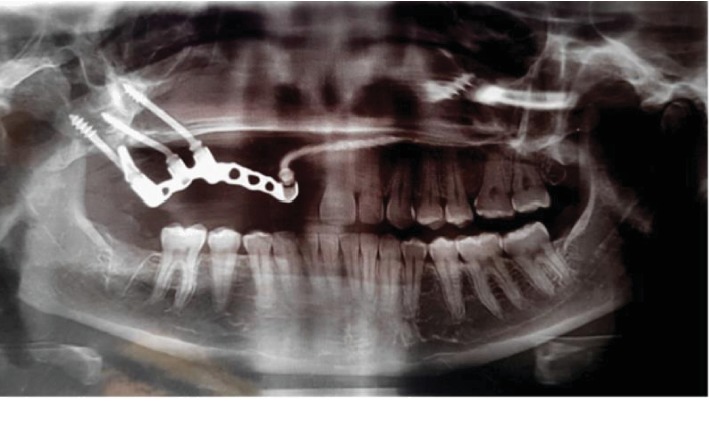
The panoramic view of the patients after 4 years of function showing an increased bone-implant contact without peri-implant radiolucency.

The patient was scheduled for follow-up after 1 week, and 3, 6, 9, 12, and 18 months and 6 months, therefore. At each follow-up, the patient was examined both clinically and radiographically. She had no complaints, and her speech, mastication, and esthetics had improved significantly. She was highly satisfied with the result and had become married. After 4 years of function, the patient presented with an optimum peri-implant health, a stable prosthesis without complaints, and a high satisfaction level. The panoramic view showed an increase in bone-implant contact and no evidence of peri-implant radiolucency (Figure 8). The patient gave the investigators a signed consent for the publication of this case.

## 3. Discussion

The primary objective of maxillary resection rehabilitation is the restoration of the patient's previous appearance and function [2–5]. Procedure success depends on both the dentist's judgment and skill, and the postoperative anatomical, physiological, and psychological condition of the patient.

The function of obturator prostheses is directly influenced by the location and size of the maxillectomy defect and the quality and quantity of the remaining teeth, soft, and bone tissues [2–4].

Even though numerous advances in surgical reconstruction have been documented, it is not always possible because of the general health condition of the patient, defect size, probability of tumor recurrence, and patient preference. In such situations, prosthetic rehabilitation is considered the treatment of choice [8].

In the present case, the nature of the tumor and the patient preference limited the immediate surgical reconstruction approach; only soft approximation was encountered [22]. Although this approximation reduced the size of the defect and closed the oroantral communication, it adversely affects the final prosthesis's retention, stability, and support. Bidra et al. [12] reported that sometimes both the bulky and flaccid nature of the remaining tissue preclude conventional prosthesis support; therefore, other means of support should be used, such as implant therapy.

Many authors [7–16] reported the successful use of the pterygoid and zygomatic implants for cases of severely resorbed maxilla and maxillary defects reconstruction. Both implants offered the utilization of the thick cortical bone for implant anchorage and increased the possibility of bicortical or even tricortical anchorage, it eliminates donor-site morbidity and/or graft material infection [17].

According to the literature, technical difficulty, peri-implant soft tissue infection, sinusitis, and fracture of the prosthetic veneer are the common complications reported with zygomatic implants [8]. These complications may have been related to the characteristics of the implant surface, the implant-abutment connection, the surgical procedure, occlusal overload, and implant's micromovement during functioning [8].

Corticobasal implants specially BCs® are one-piece implants characterized by a thin penetrating tip ensuring quick soft tissue healing, smooth polished surface improving the peri-implant soft tissue health [17, 18], isoelastic prosperity offering implant bending without compromising its survival [19, 21], and implants splinting with a supporting metal framework for better force distribution and to counteract the implant length cantilever [17, 19]; the horizontal plates of the implants are deeply anchored inside the basal bone (osseofixated) with high stability reducing the possibility of micromovement [17–19]. Such features, in addition to the hygienic extension of the prosthesis, denture base justified the use of these implant-supported prosthesis in this present case with a susceptible high success rate.

The 4 years follow-up of this clinical report is an encouraging result as according to the systemic review conducted by Goiato et al. [8]; they studied 1541 zygomatic implants among which 33 implants failed; they reported that failure generally occurred during the first-year interval. In our knowledge, this is the first study reporting the use of basal implant in a patient with maxillectomy.

Despite the success rate reported in this case being in line with the other endosseous implant's literature [8, 15, 16], a longitudinal prospective study with a large sample should be considered in the future to fill the gap of knowledge.

## 4. Conclusion

This clinical case highlighted the feasibility of basal implant-supported prosthesis as a successful treatment modality for patients with subtotal maxillectomy after soft tissue closure. It restored the patient's masticatory function, esthetics, and phonetics and improved her self-esteem and quality of life.
